# Creation of a planned or central-clefted puncture combined with a second puncture during vertebroplasty to treat osteoporotic vertebral compression fractures with large clefts

**DOI:** 10.1186/s13018-020-02048-z

**Published:** 2020-11-16

**Authors:** Chi Li, Yang Zhou, Min-yu Zhu, Yu Wang, Zheng-mao Zhang, Hong-lin Teng, Jing Wang

**Affiliations:** 1 Department of Orthopaedics (Spine Surgery), The First Affiliated Hospital of Wenzhou Medical University, Wenzhou, Zhejiang 325000 China; 2 Department of Orthopaedics, Yuhuan County People’s Hospital, Taizhou, 317600 China

**Keywords:** Osteoporotic fractures, Osteonecrosis, Vertebroplasty, Punctures

## Abstract

**Background:**

Cemented vertebrae frequently re-fracture after vertebroplasty to treat osteoporotic vertebral compression fractures (OVCFs) with large clefts. We compared the efficacy of planned and central-clefted puncture, both followed by a second puncture, as treatments for OVCFs with large clefts.

**Methods:**

We retrospectively studied 38 patients. 18 of whom underwent planned puncture (group A) and 20 central-clefted puncture (group B). A second puncture was performed when the initially injected cement was restricted to the cleft. We recorded a visual analog scale (VAS) pain scores, vertebral kyphotic angles (KAs), and compression ratios (CRs) preoperatively and at 2 days and 6 months postoperatively. We recorded the cement dispersion patterns and complications.

**Results:**

Second punctures succeeded in 15/18 and 7/20 patients of groups A and B, respectively. At 2 days postoperatively, the VAS score, KA, and CR were significantly better than the preoperative values (*P* < 0.01); no significant difference was found between the two groups (*P* > 0.05). At the 6-month follow-up, all scores were poorer than at 2 days postoperatively (all *P* < 0.05), significantly more so in group B than group A (*P* < 0.05). Significant differences in terms of the cement dispersion patterns, and the cemented vertebral re-fracture and cement leakage rates, were observed between the two groups (all *P* < 0.05).

**Conclusion:**

The two-puncture techniques were initially effective when treating large-clefted OVCFs. However, compared to the central-clefted puncture, the planned puncture improved the success rate of the second puncture, allowed better cement dispersion, and reduced the incidence of vertebral re-fracture during follow-up.

## Introduction

Percutaneous vertebroplasty usually effectively treats osteoporotic vertebral compression fractures (OVCFs) with clefts [1, 2]. However, recurrent back pain caused by re-fracture is not uncommon [3–6]; a cleft in the OVCF combined with poor cement dispersion within the fractured vertebra are the two prime risk factors [4, 6, 7]. In patients with large-clefted OVCFs (cleft height > 50% of the affected vertebral height), interdigitating cement dispersion is technically challenging because the large cleft requires more cement to fill; less cement diffuses outside the cleft. Cement dispersion during initial vertebroplasty must be improved [8]. Repeat needle insertions [9, 10] or punctures [2] have been described. However, cement dispersion remained unsatisfactory during initial vertebroplasty. Occasionally, the second puncture failed because the passage to the uncemented cancellous bone was obstructed by hardened cement in the cleft. Therefore, we developed a new puncture strategy. To the best of our knowledge, no previous report has used both a planned and a second puncture to improve cement dispersion.

## Materials and methods

### Patient selection

The study was approved by our Institutional Review Board. We enrolled 38 patients who underwent vertebroplasty to treat single-level, large-clefted OVCFs from January 2015 to June 2019. The inclusion criteria were an OVCF with a large cleft (cleft height on the computed tomography [CT] sagittal reconstruction > 50% of the fractured vertebral height evident in the prone position), increased back pain on the application of digital pressure to the spinous process of the involved vertebra, and the cleft sign evident on CT and magnetic resonance imaging [11]. The exclusion criteria were severely compressed vertebrae (complete loss of central vertebral body height) and cases who did not require second punctures, neoplastic fractures, spinal infections, and OVCFs with compromised posterior vertebral walls or neurological deficiencies.

### The procedures

Patients were placed prone on a radiolucent table and the chest and pelvis supported by soft pillows. All procedures were performed under local anesthesia. Mild sedation (dexmedetomidine hydrochloride) was induced in cases that tolerated needle insertion and cement injection poorly. Vital signs were monitored closely. All procedures were performed by two spinal surgeons, each with more than 15 years of experience. Puncture needles (11-gauge) were inserted bilaterally under fluoroscopic (C-arm) guidance. Vertebroplasty was performed as described previously [12], but with modification of the direction by which the needle traveled toward the intravertebral cleft. Two small skin incisions were made with a #11 scalpel blade. A slight twisting motion was used to advance the tip through the cortex. The needle tips were placed within the soft bone marrow of the pedicle, and minimal pressure used to advance the needles. When a needle reached the posterior vertebral margin (evident on the lateral view), the tip lays inside the medial border of the pedicle on the anteroposterior view and was then advanced into the vertebral body.

**Fig. 1:**
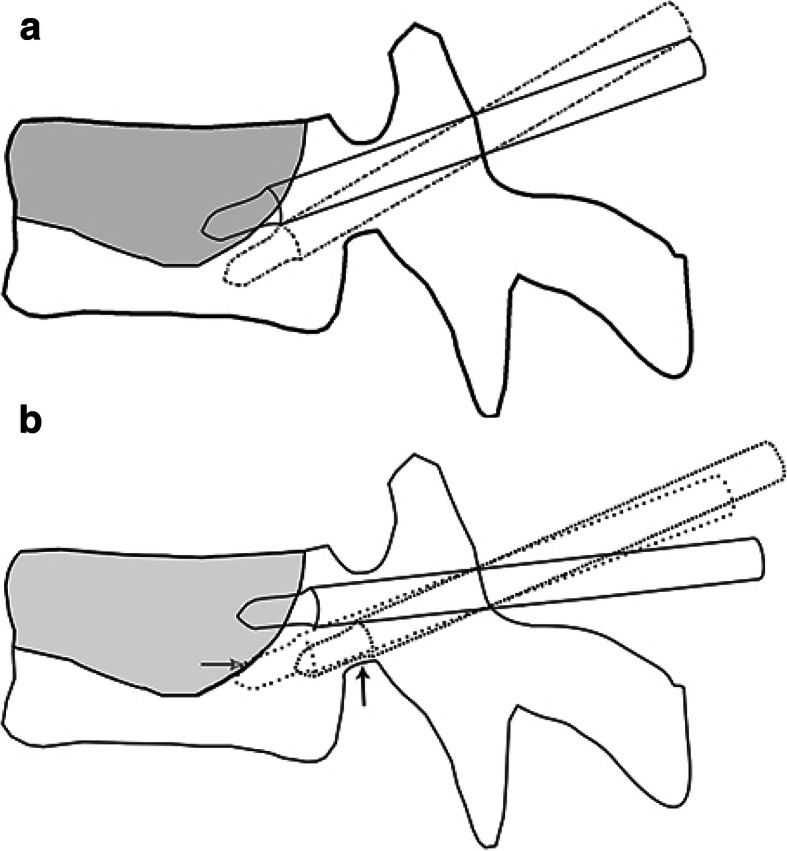
A graphic of the trajectories of the initial and second punctures. The solid line represents the initial trajectory of the puncture needle and the dotted line the trajectory of the second puncture. The intravertebral area in gray is the cement-filled cleft. **a** Planned puncture: The initial puncture trajectory lies along the border between the cleft and surrounding cancellous bone, and the needle tip lies in the cleft (near the surrounding bone) when the initial puncture is completed. When the initially injected cement is restricted to the cleft, a second puncture is made via the same pedicle entry point. **b** Central-clefted puncture: The pedicle entry point and trajectory are chosen at random. When the initial puncture is completed, the needle tip lies in the center of the cleft. Frequently, the second puncture fails because the path to the region lacking cement is blocked by the (hardened) initially injected cement (white arrow) or the lower border of the pedicle (black arrow)

For planned puncture patients (Group A), the pedicle entry points were chosen near the extension of the junction between the intravertebral cleft and peripheral cancellous bone. The needles were inserted in descending order through the pedicle and advanced to lie as close as possible to the border of the cleft and the surrounding bone. Then, the tips were positioned in the cleft near the bone (Fig. 1a).

For central-clefted puncture patients (group B), the pedicle entry point and puncture trajectory were chosen at random. When the initial puncture was completed, the needle tips were positioned in the center of the intravertebral cleft rather than at the border between the cleft and cancellous bone (Fig. 1b).

**Fig. 2:**
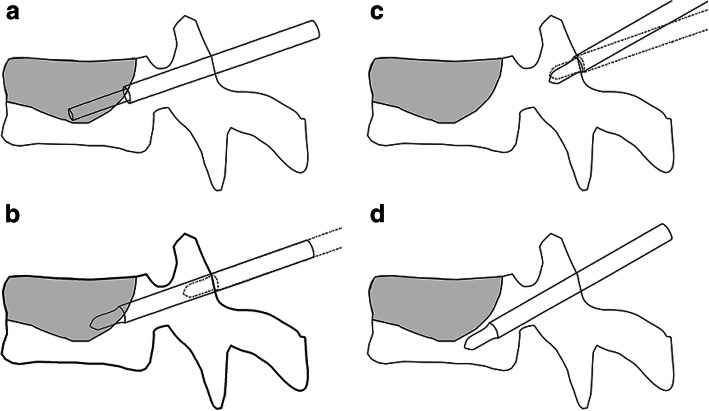
A graphic of the second puncture procedure. **a** The cement is restricted to the cleft (there is no extra-cleft dispersion) when initial cement injection through the cannula is completed. **b** The cement introducer is removed and replaced with the inner stylet (solid line). The needle is then retracted to the pedicle entry point until the tip can be redirected (dotted line). **c** At the same pedicle entry point as before, the cephalocaudal needle direction is changed to that of the region that is not filled with cement, but the abduction angle is unchanged (the dotted and solid lines show the initial and redirected needle positions, respectively). **d** The redirected needle is advanced to the intravertebral region that lacks cement

**Fig. 3:**
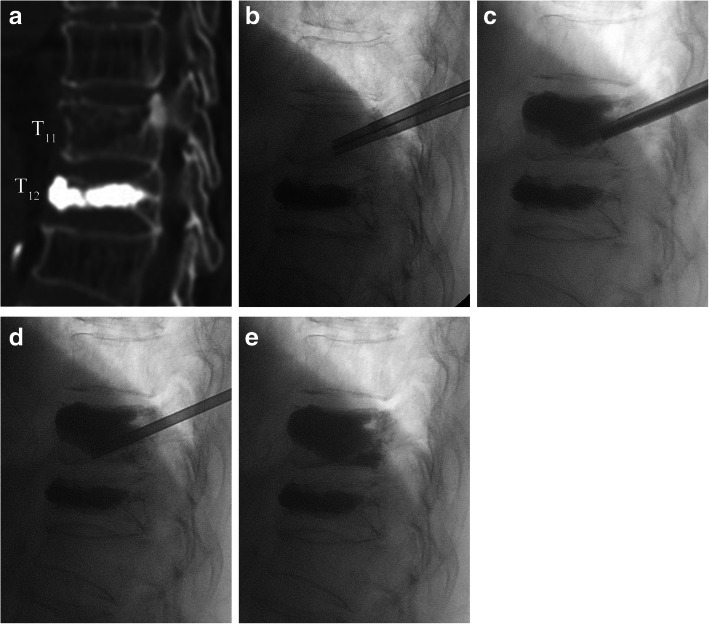
A 77-year-old female diagnosed with OVCF and a cleft underwent vertebroplasty using both a planned and a second puncture. **a** The preoperative sagittal CT reconstruction reveals a T_11_, large, irregular intravertebral cleft with little surrounding the cancellous bone, and the fact that T_12_ had undergone vertebroplasty 1 year prior. **b** The trajectory of the initial needle puncture lay along the junction between the cleft and peripheral cancellous bone and thus could be easily adjusted if a second puncture was necessary. **c** After initial cement injection, the cement was restricted to the cleft. **d** Redirecting the needle to the region lacking cement. **e** Final cement injection featuring intra- and extra-cleft dispersion

The inner stylets were removed, and cement injected through the cannula until a pressure endpoint was reached. If cement leakage occurred, the needle tip was positioned elsewhere along the initial trajectory and cement injection continued. A second puncture was created when cement was restricted to the cleft after initial injection (Fig. 2a). The cement introducer was removed and replaced with the inner stylet (Fig. 2b). The needle was withdrawn to the same pedicle entry point and the cephalocaudal direction changed (Fig. 2c). The redirected needle (thus with a new cephalocaudal angle but the same abduction angle) was advanced to the region that lacked cement (Fig. 2d). After the needle tip position was fluoroscopically confirmed, cement was injected. No patient underwent a third puncture. Figure 3 shows a case featuring a planned and a second puncture. All patients were encouraged to walk on the second postoperative day. Back braces were applied for 1 or 2 months. Osteoporotic medications, including bisphosphonates, vitamin D, and calcium, were prescribed postoperatively.

### Evaluation of clinical parameters

Patient sex, age at surgery, bone mineral density (BMD), visual analog scale (VAS), and back pain scores were recorded. BMD was measured via dual-energy X-ray absorptiometry. The second puncture success rate, the number of fluoroscopic views, and the cement injection volumes were recorded.

### Radiological assessment

**Fig. 4:**
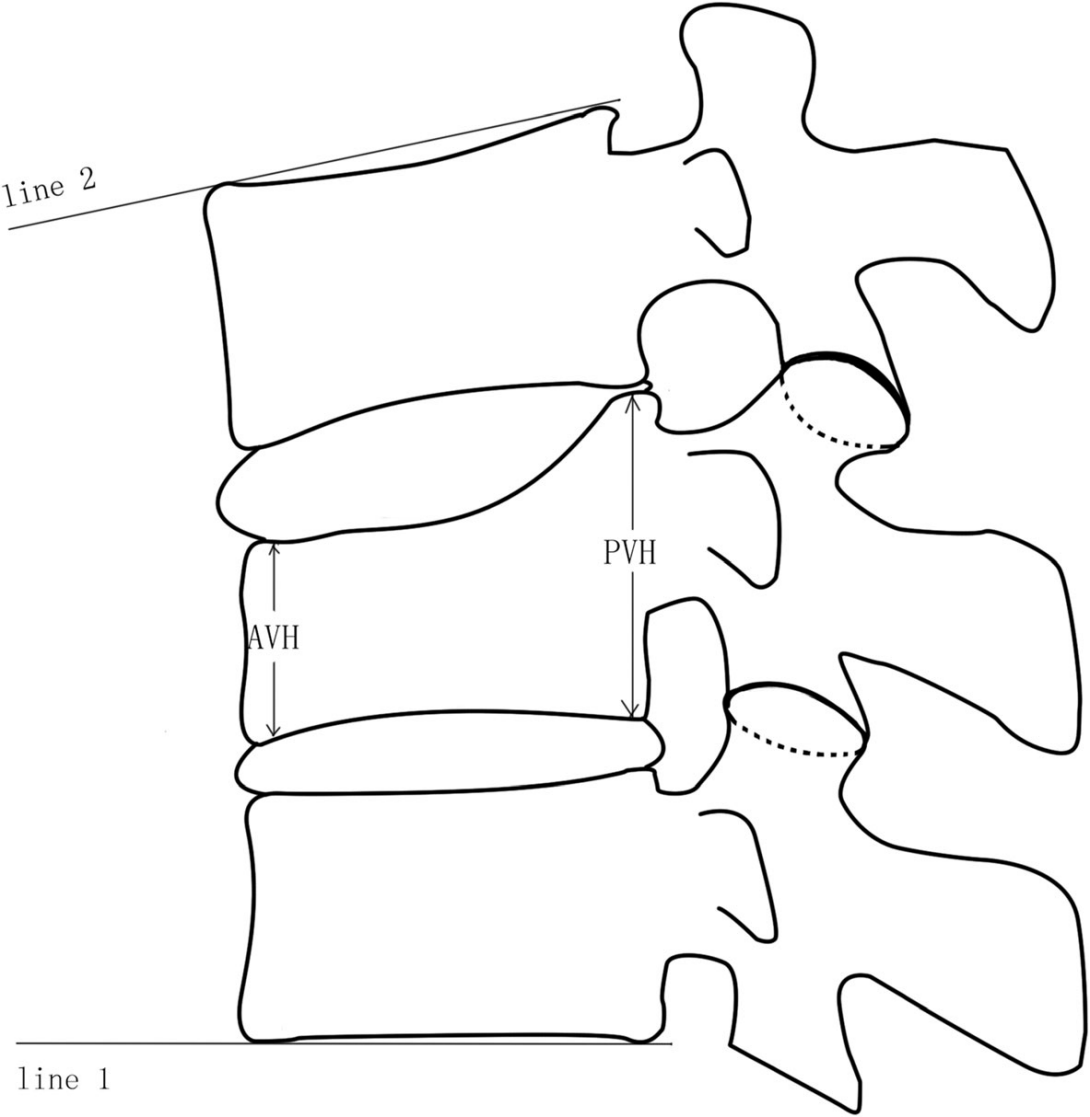
The methods used to derive measurements from lateral radiographs. KA (kyphotic angle) = the angle between line 1 and line 2. CR (compression ratio) = AVH/PVH. AVH anterior vertebral height, *PVH* posterior vertebral height

**Fig. 5:**
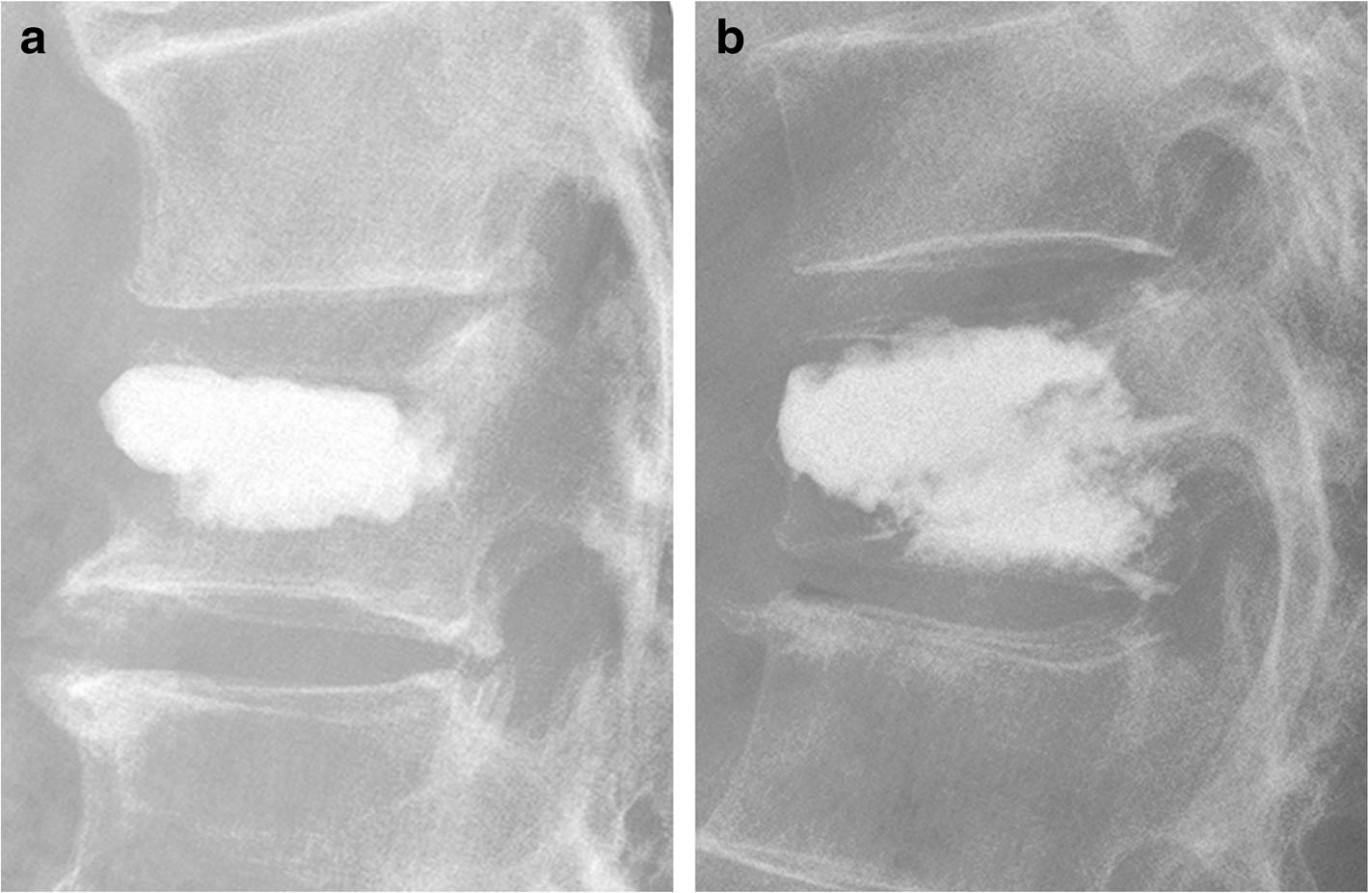
The patterns of cement distribution. **a** The cleft-filling pattern. **b** The interdigitated pattern

Images were obtained with patients supine before and after vertebroplasty. The vertebral kyphotic angle (KA) and compression ratio (CR) were measured on PACS monitors by two experienced radiologists. As shown in Fig. 4, the KA was measured as described by Kim et al. [7], using the Cobb method to evaluate vertebrae adjacent to the affected vertebra. The CR was the percentage of the anterior vertebral height (AVH) in terms of the posterior vertebral height (PVH) [13]. The cement dispersion patterns, cemented vertebral re-fractures, and cement leakages were recorded. Cement dispersion patterns were categorized (based on postoperative fluoroscopic images) as cleft-filling (compact solid cement restricted to the cleft (Fig. 5a) or interdigitated (cement filled the cleft and infiltrated the surrounding cancellous bone (Fig. 5b). A cemented vertebral re-fracture was diagnosed when recurrent back pain was accompanied by height loss or kyphosis re-occurrence was evident radiologically [3].

### Statistical analysis

SPSS ver. 20.0 statistical software (IBM Corp., Armonk, New York, USA) was used for all analyses. We report means ± standard deviations. Quantitative data were checked in terms of the normality of distribution. If the distribution was normal, ANOVA or the *t* test was used for comparisons; if not, the Mann-Whitney *U* test was employed. The *χ*^2^ test was used to compare categorical variables. The paired *t* test was employed to compare parameters at two times; thus, the preoperative to the postoperative day 2 data and the latter data to the 6-month data. A *P* value < 0.05 was considered statistically significant.

## Results

The mean patient age was 67.89 ± 6.29 years and the male/female ratio 8/30. The mean BMD T-score was 3.20 ± 0.51. Patient characteristics are listed in Table 1.

**Table 1:** Demographic and baseline characteristics

	Group A (*n* = 18)	Group B (*n* = 20)	*P*
Age (years)	69.31 ± 5.98	66.75 ± 6.45	0.230
Sex (male/female)	3/15	5/15	0.441
BMD *T* score	3.29 ± 0.46	3.13 ± 0.54	0.341
Treated vertebrae level			0.096
T11	2	0	
T12	8	9	
L1	4	10	
L2	4	1	

Values are presented as number or mean ± SD

*BMD* bone mineral density

### Clinical findings

The clinical data for all patients are listed in Table 2 and the group data in Table 3. The VAS pain scores (both groups) decreased significantly on postoperative day 2 (compared to the pre-operative data) (*P* < 0.01); the groups did not differ (*P* > 0.05). At 6 months postoperatively, the VAS scores had increased significantly compared to the postoperative day 2 scores (*P* < 0.05), significantly more so in group B than A (*P* < 0.05). The second punctures succeeded in 15/18 patients of group A and 7/20 of group B (*P* < 0.01). The total fluoroscopic views numbered 17.56 ± 3.34 in group A and 13.55 ± 2.64 in group B (*P* = 0.01). The cement volume differed significantly between the groups (8.34 ± 0.81 mL for group A and 7.00 ± 0.93 mL for group B) (*P* < 0.01). The average volume of second-injection cement was 1.18 ± 0.47 mL.

**Table 2:** Clinical and radiologic indicators at 3 time points

	Preoperative	Postoperative 2 days	Postoperative 6 months	*P*_1_	*P*_2_
VAS	7.08 ± 0.91	2.82 ± 0.56	3.39 ± 1.36	0.000	0.023
KA (°)	22.22 ± 8.50	16.04 ± 6.23	20.50 ± 7.23	0.000	0.000
CR (%)	61.18 ± 13.12	88.65 ± 13.62	74.89 ± 13.40	0.000	0.000

*P*_1_ represents the preoperative time point vs. postoperative day 2, and *P*_2_ represents the postoperative day 2 vs. postoperative 6 months

*VAS* Visual analog scale, *KA* Kyphotic angle, *CR* Compression ratio

**Table 3:** Clinical and radiologic indicators between groups

	Group A	Group B	*P*
VAS			
Preoperative	7.11 ± 0.75	7.05 ± 1.05	
Postoperative 2 days	2.72 ± 0.46	2.90 ± 0.64	0.534*
Postoperative 6 months	2.72 ± 0.89	4.00 ± 1.45	0.044**
KA (°)			
Preoperative	23.82 ± 6.26	21.69 ± 10.25	
Postoperative 2 days	15.55 ± 5.56	16.48 ± 6.89	0.158*
Postoperative 6 months	18.26 ± 5.30	22.52 ± 8.23	0.024**
CR (%)			
Preoperative	60.44 ± 11.52	61.88 ± 14.67	
Postoperative 2 days	90.40 ± 13.60	85.62 ± 13.57	0.088*
Postoperative 6 months	82.01 ± 10.76	68.49 ± 12.44	0.029**

*VAS* Visual analog scale, *KA* Kyphotic angle, *CR* Compression ratio

*Preoperative vs. postoperative day 2

**The postoperative day 2 vs. postoperative 6 months

### Radiological findings

Radiological data for all patients are listed in Table 2; those for the groups are listed in Table 3. The CR and KA were significantly corrected on day 2 postoperatively compared to the preoperative data (*P* < 0.01); the two groups did not differ (*P* > 0.05). At the 6-month follow-up, all CR and KA values were poorer than the postoperative day 2 data (*P* < 0.01), more so in group B than group A (*P* < 0.05). The cleft-filling cement dispersion pattern was found in 3/18 vertebrae of group A and 11/20 vertebrae of group B; the interdigitated pattern was observed in 15/18 and 9/20 vertebrae of groups A and B, respectively (*P* < 0.05).

### Complications

Cement leakage was observed from 10/18 vertebrae in group A and 4/20 vertebrae in group B (*P* < 0.05). Small amounts of cement leaked into the epidural space (two patients), paravertebral soft tissue (three), and intervertebral disc space (nine). No fatal cement-related complication (a pulmonary embolism or a neurological deficiency) was noted. Cemented vertebral re-fractures were observed in 2/18 vertebrae of group A and 10/20 of group B, thus significantly more often in group B (*P* < 0.05). Three patients underwent pedicle screw fixation later than 6 months postoperatively; the others received non-surgical treatments and two developed intervertebral bridging ossifications during follow-up.

## Discussion

An intravertebral cleft can render a fractured vertebra dynamically mobile [14]. Patients with clefts usually have severe back pain and are refractory to conservative treatments including bed rest and medication [15]. Histological studies have confirmed that clefts are composed of necrotic cancellous bone, hyaline cartilage, fractured callus, and fluid [16]. Cleft-filling with cement restores spinal stability and relieves pain [1, 17]. However, the membrane around the cleft prevents cement interdigitation with surrounding cancellous bone; the cement becomes a solid lump, greatly stressing the (weakened) surrounding cancellous bone, causing the collapse of the “non-cement-supported” area [4]. Therefore, re-fracture of a previously treated vertebra is common in clefted OVCF patients [4, 7].

Successful treatment of OVCFs with clefts requires that the intra-cleft cement injected both fills the cleft and interdigitates with surrounding cancellous bone [18]. If the cleft is small, interdigitated cement diffusion after a puncture is good, because cleft filling per se requires little cement; any further cement injection could breach the cleft boundary. However, after intra-cleft puncture of a large-clefted OVCF, more cement is required to fill the cleft, leaving less to interdigitate within the peripheral cancellous bone. When a second puncture is needed to inject more cement, it can be difficult to adjust the needle tip because the (hardened) cement of the initial injection blocks a second puncture, or the uncemented extra-cleft space is inaccessible via any trajectory commencing at the initial pedicle entry point. Solid intra-cleft cement dispersions are sometimes found in patients with large clefts who had undergone central-clefted punctures. He [8] found that patients exhibiting such dispersions had a higher incidence of cemented vertebral re-compression than did patients in whom the cement had interdigitated, as we also observed. Few OVCFs with large clefts were described in previous reports [19], but were associated with most cases of cemented vertebral re-fracture [2–4, 19, 20]. Vertebroplasty of a large (compared to a small)-clefted OVCF is technically more challenging.

Many factors affect cement dispersion within a vertebral body [21]. Most clinical studies have used punctures to improve cement dispersion. Chen et al. [10] used a Kirschner wire-guided technique to insert a second needle via the initial pedicle entry point. However, repeat needle insertion sometimes failed if the second puncture trajectory was blocked by hardened cement. A new pedicle entry point has been used for repeat puncture [9], but this is surgically time-consuming; also, sometimes, a new point cannot be chosen because, radiographically, the pedicle is obscured by the initially injected cement. Yu et al. [2] used a puncture technique to treat clefted OVCFs. The needle tip was positioned in extra-cleft cancellous bone, and a balloon inflated to compact the surrounding bone until the periphery of the cleft was broken. However, the technique had a drawback. Patients often present with both severe osteoporosis and minimal cancellous bone around the cleft. In OVCF patients with large clefts, the balloon compresses the remaining cancellous bone until that bone breaks sclerotic bone around the cleft, thus rendering it difficult to interdigitate cement into the surrounding cancellous bone and increasing the risk of cement dislodgement.

A second cement injection is often needed when the initially injected cement is restricted to the cleft. However, occasionally, the second injection fails when the (hardened) cleft-filling cement or the chosen pedicle entry point render a new trajectory to the uncemented intravertebral region impossible. Therefore, our initial puncture lay along the junction between the cleft and the surrounding cancellous bone, so that the needle could be redirected to a region lacking cement, employing the same pedicle entry point, more easily than when a central-clefted puncture was employed. To the best of our knowledge, this is the first use of a planned-puncture-with-second-puncture strategy to treat clefted OVCFs. We found that both puncture strategies reduced vertebral re-fracture and relieved back pain instantly. Compared to central-clefted puncture; however, planned puncture was associated with a higher success rate of second puncture and a more interdigitated cement distribution postoperatively. Furthermore, less correction loss and a lower re-fracture rate of cemented vertebrae were evident in the planned puncture group at the 6-month follow-up, although more fluoroscopic views were required when using this approach. Given the high incidence of cemented vertebral re-fracture in OVCF patients with clefts (as we and others [4, 7] report), the combination of a planned and second puncture is recommended, despite the increased radiation exposure.

Another complication was cement leakage. Patients with intravertebral clefts, cortical disruptions, and those who receive high volumes of injected cement may be at risk of leakage [22]. To correct and maintain the fractured vertebrae, more cement was injected in group A than group B, perhaps explaining the high leakage rate in group A. However, we encountered no fatal cement-related complication.

Our work had certain limitations. First, the work was retrospective in nature and the sample size small; OVCF cases with large clefts facilitating vertebroplasty are rare. Also, the follow-up duration was short, because either the surgery per se or delayed intervertebral bridging ossification in some patients with cemented vertebral re-fractures affected the outcomes later than 6 months postoperatively. A prospective study with a larger sample size and longer follow-up is needed. Second, “large clefts” are not defined. We observed a high incidence of cemented vertebral re-fracture in OVCF cases with what we considered were large clefts, as also reported in previous studies [2–4, 19, 20]. A consensus definition of a “large cleft” is required.

In conclusion, both puncture strategies were initially effective when treating OVCFs with large clefts. However, vertebrae cemented via central-clefted puncture frequently re-fractured. A planned puncture increased the success rate of the second puncture and improved cement dispersion, reducing re-fracture. We recommend the use of planned puncture during vertebroplasty to treat OVCFs with large clefts.
